# Efficacy of music intervention on pain and anxiety in patients undergoing cataract surgery: a systematic review and meta-analysis

**DOI:** 10.3389/fpsyt.2025.1600359

**Published:** 2025-06-19

**Authors:** Lanzhu Su, Yuanyuan Zhang, Lixin Lu

**Affiliations:** Department of Ophthalmology, Beijing Tongren Eye Center, Beijing Tongren Hospital, Capital Medical University, Beijing, China

**Keywords:** music therapy, VAS, STAI, SAS, meta-analysis

## Abstract

**Objective:**

To evaluate the effectiveness of music therapy in reducing anxiety and pain among patients undergoing cataract surgery.

**Methods:**

Relevant studies up to May 2024 were identified by searching PubMed, Embase, Cochrane, Web of Science, CNKI, and Wanfang databases. Literature selection followed PICOS criteria, with methodological quality assessed using the Cochrane risk of bias tool. Meta-analysis employed standardized mean differences (SMD). Sensitivity and subgroup analyses explored result stability and heterogeneity, utilizing Review Manager 5.4 and STATA 15.0 for analysis.

**Results:**

Eighteen studies with 2,262 participants were included. Music therapy significantly reduced anxiety levels, as demonstrated by a notable decrease in Visual Analog Scale (VAS) anxiety scores (SMD = -7.10, 95% CI: -12.25 to -1.95) and State-Trait Anxiety Inventory (STAI) scores (SMD = -1.26, 95% CI: -1.85 to -0.66). Self-Rating Anxiety Scale (SAS) scores were also significantly lower in the music therapy group (SMD = -0.27, 95% CI: -0.42 to -0.12). Regarding physiological parameters, music therapy significantly reduced systolic blood pressure (SBP) (SMD = -0.58, 95% CI: -0.80 to -0.35), diastolic blood pressure (DBP) (SMD = -0.27, 95% CI: -0.42 to -0.12), and heart rate (HR) (SMD = -0.31, 95% CI: -0.45 to -0.17). Subgroup analysis indicated greater therapeutic efficacy among Asian populations compared to European populations.

**Conclusion:**

Music therapy significantly reduces anxiety and pain in cataract surgery patients and improves vital signs to some extent. However, due to heterogeneity in certain results, further high-quality randomized controlled trials (RCTs) are needed to confirm its effectiveness.

**Systematic review registration:**

https://www.crd.york.ac.uk/prospero/, identifier CRD42024586504.

## 1 Introduction

Cataract is defined as the clouding of the lens in the eye, which leads to progressive vision impairment and remains the leading cause of blindness globally (1).

Cataracts are the leading cause of blindness worldwide. According to the Global Burden of Disease (GBD) study, approximately 17 million people globally were blind due to cataracts in 2020, and 83.5 million suffered from moderate to severe visual impairment caused by this condition (2). Cataracts, a common ophthalmological disease, are increasingly prevalent with the aging population, posing a significant public health challenge to vision (3). Elderly cataract patients often exhibit marked preoperative anxiety, which tends to be more pronounced compared to younger patients (4). This anxiety primarily arises from the requirement to remain awake under local anesthesia during surgery, maintaining calmness and stillness while strictly following the instructions of medical staff (5). These unique surgical demands can be particularly challenging for anxious patients, often manifesting as physiological responses such as tachycardia, hypertension, and hyperventilation (6).

Music is regarded as a safe, cost-effective, non-pharmacological intervention and is widely applied across various fields (7). Merakou et al. (8) suggest that music has a unique relationship with cardiovascular health, as carefully selected music can promote relaxation, reducing respiratory rate, heart rate, and arterial blood pressure. Cruise et al. (9) reported that music interventions significantly decreased the need for sedatives in patients undergoing orthopedic and plastic surgeries under local anesthesia. Additionally, studies found that listening to music reduced blood pressure and induced a more relaxed emotional state in plastic surgery patients (10). These findings highlight the potential of music as an effective non-pharmacological approach to improving both physiological and psychological conditions in surgical patients.

The rationale for using music during cataract surgery is multifaceted: it can mask anxiety-provoking surgical sounds, provide a familiar and comforting stimulus in an unfamiliar environment, and potentially reduce the need for pharmacological sedation (11).

In cataract surgery, music interventions typically involve playing music before or during the procedure to help patients relax and alleviate anxiety (12).

This study aims to systematically review and conduct a meta-analysis to investigate the effects of music on patients undergoing ophthalmic surgery, with a particular focus on its application in cataract surgery. Although randomized controlled trials (RCTs) have demonstrated that music interventions effectively alleviate anxiety in cataract surgery patients, there remains a lack of robust evidence-based support to comprehensively validate these findings. Therefore, further high-quality research is essential to strengthen the evidence base in this field and clarify the clinical benefits of music interventions.

## 2 Materials and methods

### 2.1 Study design

This study follows the PRISMA guidelines to analyze the effects of music interventions on cataract surgery patients (13). The study has been prospectively registered with the International Prospective Register of Systematic Reviews (PROSPERO) (No. CRD42024586504) (14).

### 2.2 Literature search strategy

Databases including PubMed, Embase, Web of Science, Cochrane, Wan fang, and CNKI were searched, and the references of included studies were also traced as supplementary sources. The search period covered from database inception to May 2024. Key search terms included: “Music,” “Cataract,” “Surgical Procedures,” and “Operative.” Detailed search strategies are provided in **Supplementary Table S1**.

### 2.3 Inclusion and exclusion criteria

Inclusion criteria:

Study type: RCTs;
Participants: Individuals diagnosed with cataracts based on clinical symptoms such as progressive vision loss and blurred vision, along with slit-lamp examination showing lens opacity, excluding other causes of visual impairment;
Intervention: The experimental group received music intervention during the standard surgical process, while the control group received care without music. Common music types included classical, light, ethnic, and popular music, primarily with a soothing rhythm and moderate volume (40–60 dB);
Outcome measures: Anxiety scores (Self-Rating Anxiety Scale [SAS]), heart rate (HR), Visual Analog Scale (VAS), systolic blood pressure (SBP), diastolic blood pressure (DBP), and State-Trait Anxiety Inventory (STAI).

Exclusion criteria:

Irrelevant studies;
Non-English or non - Chinese literature;
Studies where full text or data was unavailable;
Duplicate publications;
Non-original research;
Literature reviews.

Studies where patients received sedative medications that could affect physiological parameters such as blood pressure and heart rate, to avoid potential confounding effects on the outcomes.

### 2.4 Literature screening and data extraction

Two researchers independently screened the studies. In case of discrepancies, a third-party opinion was sought. The extracted data included the author, year, country, sample size, SAS score, HR, VAS score, SBP, DBP, STAI, and intervention and control measures.

### 2.5 Assessment of literature quality

The quality of the included studies was assessed according to the Cochrane Handbook version 5.1.0 RCT risk of bias criteria. The risk of bias was evaluated based on seven domains: random sequence generation, allocation concealment, blinding of participants and researchers, blinding of outcome assessors, completeness of outcome data, selective reporting, and other potential biases. Each domain was rated as “low risk,” “high risk,” or “unclear.” Two researchers independently assessed the studies based on these criteria, and in case of disagreement, a third researcher was involved in the discussion to reach a consensus.

### 2.6 Statistical analysis

Literature quality assessment was conducted using Review Manager 5.4.1, and a risk of bias graph was generated. Statistical analyses of outcome measures were performed using mean difference (MD) or standardized mean difference (SMD) as effect sizes, with 95% confidence intervals (CI) calculated. Heterogeneity was assessed using the Cochrane Q test and I² statistic, where P < 0.1 or I² > 50% indicated significant heterogeneity. A random-effects model was used to pool the data.

Additionally, sensitivity analysis and subgroup analysis were performed to explore the stability of the results and potential sources of heterogeneity. Publication bias was assessed using funnel plots and Egger’s test. A significance level of α = 0.05 was set for all analyses. All statistical analyses were conducted using Review Manager 5.4.1 and STATA 15.1.

## 3 Results

### 3.1 Literature selection

**Figure 1:**
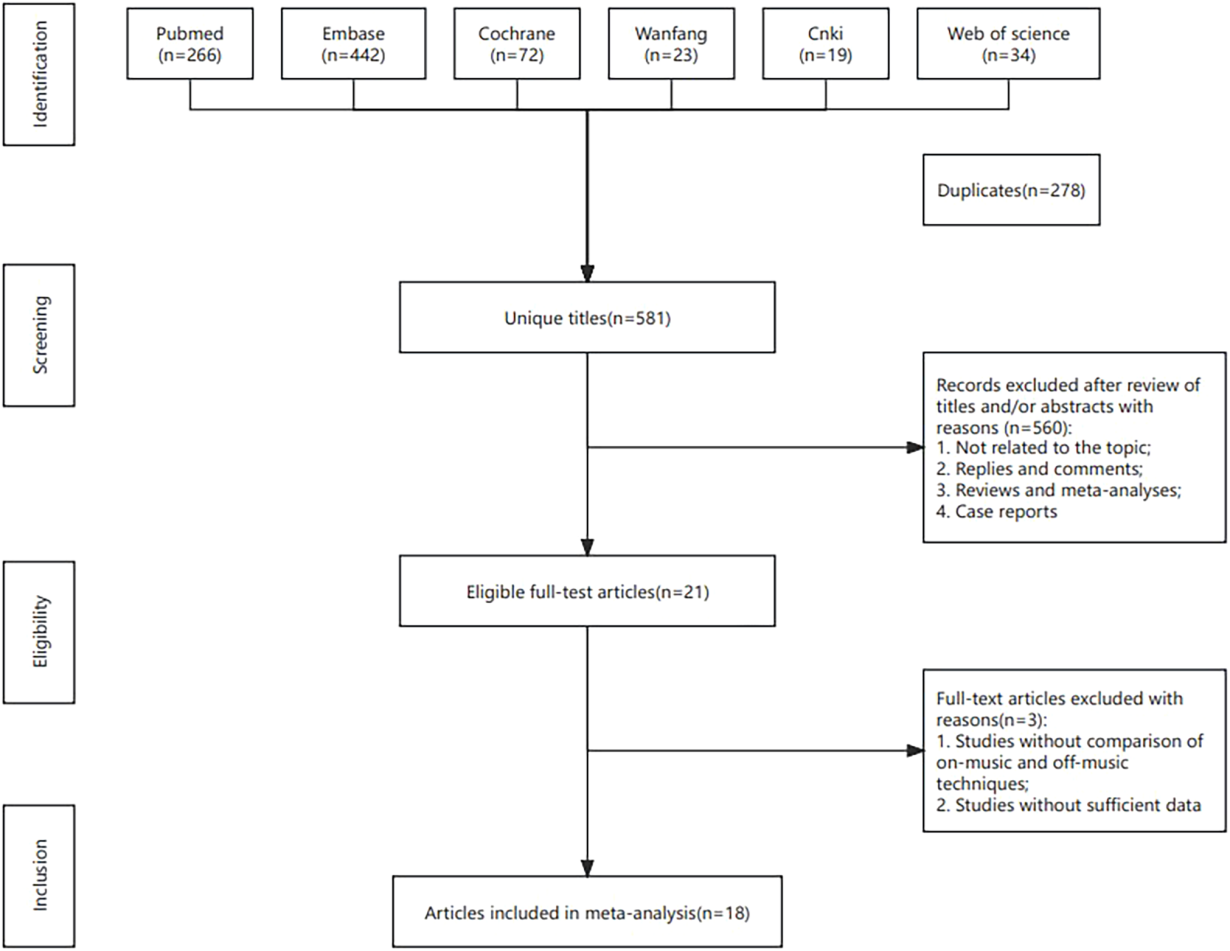
Flowchart of the systematic search and selection process.

A total of 859 studies were screened, and 18 studies (8, 11, 15–30) were ultimately included. The literature selection process is illustrated in **Figure 1**.

### 3.2 Quality assessment of included studies

**Figure 2:**
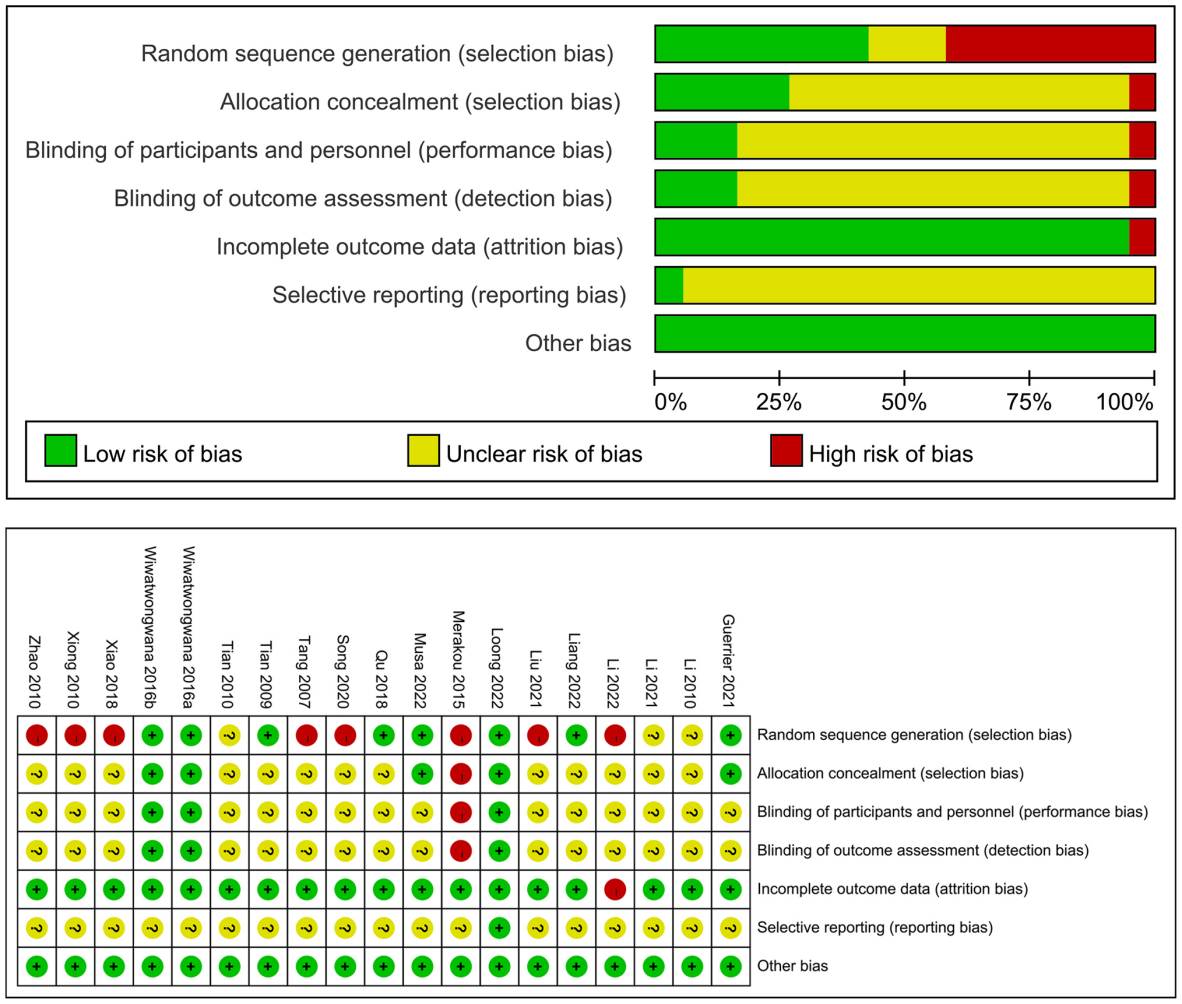
Risk of bias graph.

Among the 18 studies, 12 reported specific randomization methods, including computer-generated random lists, random number tables, and drawing lots. The remaining 6 studies only mentioned randomization but did not specify the method. Four studies described allocation concealment. One study employed blinding for both patients and investigators, while 7 studies blinded outcome assessors. The remaining studies did not report on allocation concealment or blinding procedures. Detailed quality assessment results are presented in **Figure 2**.

### 3.3 Basic characteristics of included studies

A total of 18 studies (8, 11, 15–30) were included, comprising 2,262 patients. Among these, 13 were published in Chinese and 5 in English. The publication years ranged from 2005 to 2021. The intervention in the experimental group involved music therapy, while the control group received routine care.

Four studies (17, 23, 29, 30) assessed anxiety using the Self-Rating Anxiety Scale (SAS), 15 studies (8, 11, 15, 17–22, 24–29) used HR to assess anxiety, and 3 studies (11, 17, 28) utilized the VAS for anxiety evaluation. Thirteen studies (8, 15, 17, 19–22, 24–29) assessed SBP, 12 studies (8, 15, 17, 19–22, 24–27, 29) used DBP, and 5 studies (18, 21, 24, 26, 27) evaluated anxiety using the State-Trait Anxiety Inventory (STAI). Detailed characteristics are shown in **Table 1**.

**Table 1:** The basic literature characteristics.

Authors	Study period	Country	Study design	Patients (n) Off-music/ on-music	Age Off-music/ on-music	Male Off-music/ on-music	Female Off-music/ on-music	Intervention	Control	Outcome
Guerrier et al. (11)	2017.2-2018.7	French	RCT	124/119	68.5,11.2/67.3,10.4	59/56	65/63	Headphones with patient’s preferred music(n=119)	Headphones without music(n=124)	HR、VAS
Li et al. (15)		China	RCT	25/25	68,4(61-76)/69,3 (60-78)	13/12	12/13	Music was played in the operating room during procedures(n=25)	Music was not played in the operating room during procedures(n=25)	HR、SBP、DBP
Li et al. (16)	2019.11-2020.8	China	RCT	50/50	64.0,16.5(53-89)/67.0, 20.3(55-87)	25/26	25/24	The music based on Traditional Chinese Medicine’s Five Elements was played in the operating room(n=50)	No music was played in the operation room(n=50)	
Liang et al. (17)	2018.1-2019.12	China	RCT	98/98	71.24,3.32(61-79)/72.65,4.13(62-78)	46/48	52/50	According to the different conditions of patients, horn, palace, Shang, zheng, feather 5 kinds of music(n=98)	No music(n=98)	SAS、HR、VAS、SBP、DBP
Liu et al. (18)	2018.1-2019.12	China	RCT	51/51	36.71,8.35(20-60)/35.67,8.83(18-60)	33/31	18/20	Patients’ self-selected music(n=51)	No music(n=51)	HR、STAI
Loong et al. (19)	2017.4-2018.3	Malaysia	RCT	30/31	63.9,6.2/67.7,9.0	19/14	11/17	Earphones with binaural beat music(n=31)	Earphones without music(n=30)	HR、SBP、DBP
Merakou et al. (8)	2009.8-2009.11	Greece	RCT	100/100	70.9/71.4			Headphones with meditation music(n=100)	Headphones without music(n=100)	HR、SBP、DBP
Musa et al. (20)	2016.1.18-2016.12.16	Malaysia	RCT	46/46	67.70,7.760/69.04,8.086			Piano music played in the operating theater(n=46)	No music played in the operating theater(n=46)	HR、SBP、DBP
Qu et al. (23)	2017.5-2018.9	China	RCT	48/48	>65,30<65,18/>65,25/<65,23	26/31	22/17	Light music is played to the patient through a portable mobile music device (MP3 player)(n=48)	No music(n=48)	SAS
Song et al. (24)	2016.6-2018.6	China	RCT	134/134	69.57,4.34(60-78)/71.30,4.28(61-80)	55/58	79/76	Play the music through the headphones(n=134)	No music(n=134)	HR、SBP、DBP、STAI
Li et al. (22)	2019.4-2021.1	China	RCT	50/50	54.21,3.49(40-70)/54.23,3.42(41-70)	30/28	20/22	Play the glass lake(n=50)	No music (n=50)	HR、SBP、DBP
Tang et al. (25)	2005.9-2006.1	China	RCT	50/50	68.04,7.55/69.14,7.18	18/22	32/28	Play background music(n=50)	No music (n=50)	HR、SBP、DBP
Tian et al. (26)	2007.12-2008.10	China	RCT	31/28				Play the music through the headphones(n=28)	No music (n=31)	HR、SBP、DBP、STAI
Tian et al. (27)	2007.6-2008.12	China	RCT	45/45	59.81,14.37(20 – 79/59.81,14.37(20-79)	27/24	18/21	Play the music through the headphones(n=45)	No music (n=45)	HR、SBP、DBP、STAI
Wiwatwongwana 2016a et al. (21)	2011.1-2011.4	Thailand	RCT	47/44	69.0,10.0/68.4,8.2			Earphones with binaural beat embedded music group (n =44)	No music(n=47)	HR、SBP、DBP、STAI
Wiwatwongwana 2016b et al. (21)	2011.1-2011.4	Thailand	RCT	47/44	69.0,10.0/67.0,7.8			Earphones with plain music group (n =44)	No music (n=47)	HR、SBP、DBP、STAI
Xiao et al. (28)	2016.1-2017.6	China	RCT	20/20	67.8,7.8/68.2,5.1	13/12	7/8	Use your mobile phone to play your favorite music beside your pillow(n=20)	No music(n=20)	HR、VAS、SBP
Xiong et al. (29)	2009.4.16-2009.4.21	China	RCT	99/99	(52-80)/(52-80)			Play background music in the waiting room and surgery room(n=99)	No background music is played in the waiting room and surgery room(n=99)	SAS、HR、SBP、DBP
Zhao et al. (30)	2008.4-2008.6	China	RCT	66/66	44.3(28-70)/46.1(31-72)	31/25	35/41	Background music was played in the operating room(n=66)	No background music is played in the operating room(n=66)	SAS

### 3.4 Meta-analysis

#### 3.4.1 Change in SAS score

**Figure 3:**
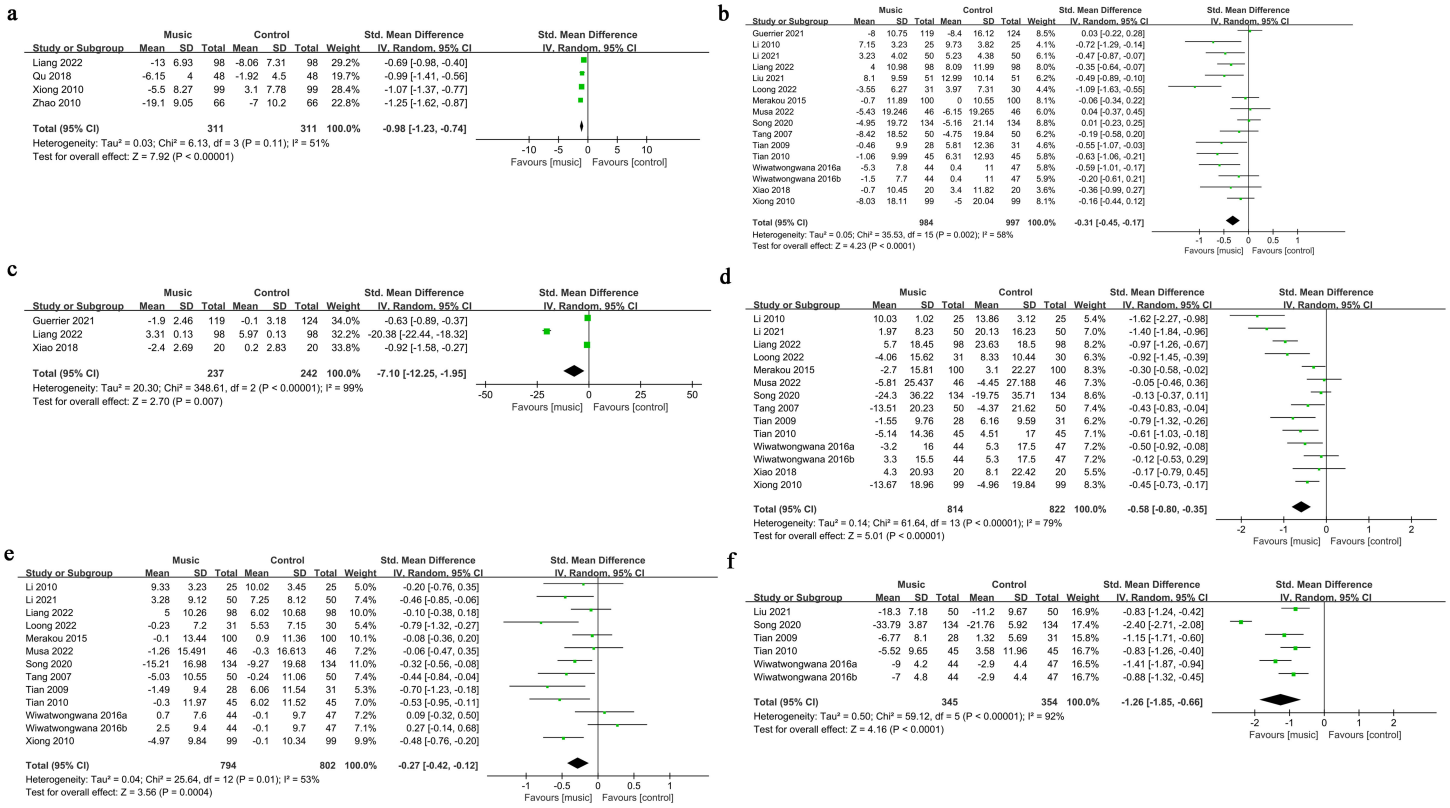
The meta-analysis results. **(a)**. Change in SAS score; **(b)**. Change in HR; **(c)**. Change in VAS score; **(d)**. Change in SBP; **(e)**. Change in DBP; **(f)**. Change in STAI.

Four studies (17, 23, 29, 30) examined the effect of music intervention on patients’ SAS scores. There was considerable heterogeneity between the studies (P = 0.01, I² = 53%), and a random-effects model was used for analysis. The meta-analysis revealed that music intervention significantly reduced SAS scores in patients (SMD = -0.27, 95% CI: -0.42 to -0.12) (**Figure 3a**).

#### 3.4.2 Change in HR

Fifteen studies (8, 11, 15, 17–22, 24–29) investigated the effect of music intervention on HR. Significant heterogeneity was observed (P = 0.002, I² = 58%), and a random-effects model was applied. The meta-analysis showed that music intervention significantly reduced HR (SMD = -0.31, 95% CI: -0.45 to -0.17) (**Figure 3b**).

#### 3.4.3 Change in VAS score

Three studies (11, 17, 28) evaluated the impact of music intervention on the VAS scores. The studies exhibited high heterogeneity (P < 0.00001, I² = 99%), and a random-effects model was used. The meta-analysis found that music intervention significantly reduced VAS scores (SMD = -7.10, 95% CI: -12.25 to -1.95) (**Figure 3c**).

#### 3.4.4 Change in SBP

Thirteen studies (8, 15, 17, 19–22, 24–29) analyzed the effect of music intervention on SBP. Significant heterogeneity was present (P < 0.00001, I² = 79%), and a random-effects model was used. The meta-analysis indicated that music intervention significantly reduced SBP (SMD = -0.58, 95% CI: -0.80 to -0.35) (**Figure 3d**).

#### 3.4.5 Change in DBP

Twelve studies (8, 15, 17, 19–22, 24–28) examined the effect of music intervention on DBP. Heterogeneity was significant (P = 0.01, I² = 53%), and a random-effects model was applied. The meta-analysis showed that music intervention significantly reduced DBP (SMD = -0.27, 95% CI: -0.42 to -0.12) (**Figure 3e**).

#### 3.4.6 Change in STAI

Five studies (18, 21, 24, 26, 27) investigated the effect of music intervention on the STAI scores. High heterogeneity was observed (P < 0.00001, I² = 92%), and a random-effects model was used. The meta-analysis revealed that music intervention significantly reduced STAI scores (SMD = -1.26, 95% CI: -1.85 to -0.66) (**Figure 3f**).

### 3.5 Publication bias detection

**Figure 4:**
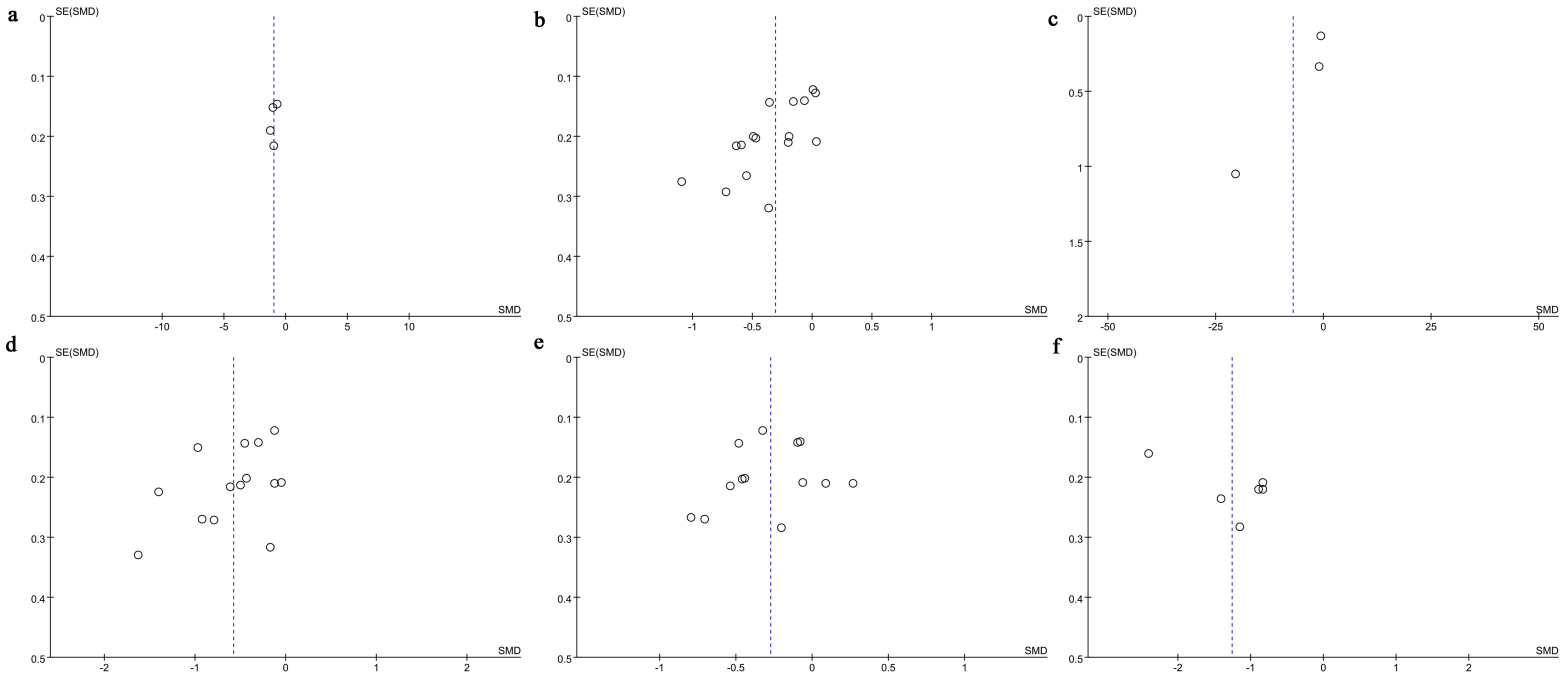
Funnel plot. **(a)** Change in SAS score; **(b)** Change in HR; **(c)** Change in VAS score; **(d)** Change in SBP; **(e)** Change in DBP; **(f)** Change in STAI.

Funnel plots revealed potential publication bias for VAS score (**Figure 4c**) and STAI (**Figure 4f**). However, no significant publication bias was observed for SAS score (**Figure 4a**), HR (**Figure 4b**), SBP (**Figure 4d**), and DBP (**Figure 4e**).

Egger’s test indicated publication bias for HR (P = 0.001), while no significant publication bias was found for SAS score (P = 0.488), VAS score (P = 0.348), SBP (P = 0.130), DBP (P = 0.566), or STAI (P = 0.102).

### 3.6 Sensitivity analysis

**Figure 5:**
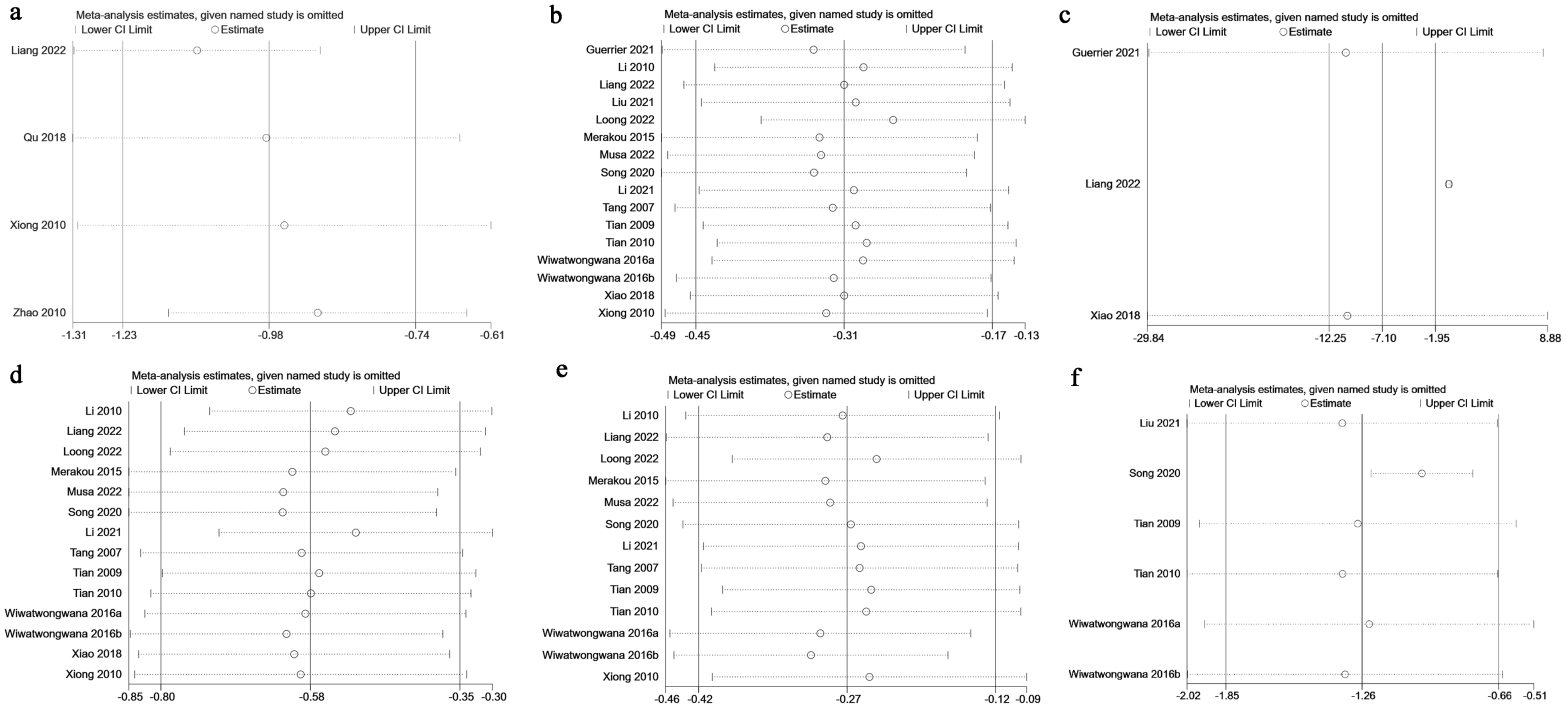
Sensitivity analysis. **(a)** Change in SAS score; **(b)** Change in HR; **(c)** Change in VAS score; **(d)** Change in SBP; **(e)** Change in DBP; **(f)** Change in STAI.

Sensitivity analysis was conducted for SAS score (**Figure 5a**), HR (**Figure 5b**), VAS score (**Figure 5c**), SBP (**Figure 5d**), DBP (**Figure 5e**), and STAI (**Figure 5f**) using a leave-one-out approach. The results indicated that for SAS score (**Figure 5a**), HR (**Figure 5b**), SBP (**Figure 5d**), DBP (**Figure 5e**), and STAI (**Figure 5f**), excluding any single study did not significantly alter the overall results, and the direction of the combined effect remained unchanged, confirming the stability of the pooled results.

However, for VAS score (**Figure 5c**), excluding the studies by Guerrier (11) and Xiao (28) led to a change from significant to non-significant results, indicating potential instability in this outcome.

### 3.7 Subgroup analysis

Subgroup analyses were performed based on sample size and geographical region for HR, SBP, and DBP. For HR, music intervention showed no significant effect in the European subgroup, while all other subgroups showed effective results. For DBP, the subgroup with a sample size smaller than 100 showed no significant effect of music intervention, and the European subgroup also yielded non-significant results. However, all other subgroups showed effective results. For SBP, music intervention was effective in all subgroups (**Table 2**).

**Table 2:** Subgroup analysis of music alleviating patient anxiety.

Subgroup	HR				SBP				DBP			
	Study	SMD [95%CI]	P value	I2	Study	SMD [95%CI]	P value	I2	Study	SMD [95%CI]	P value	I2
Total	16	0.31 [-0.45, -0.17]	<0.0001	58%	14	-0.58 [-0.80,-0.35]	<0.0001	79%	13	-0.27 [-0.42,-0.12]	0.0004	53%
Sample size												
≥100	8	-0.17 [-0.31,-0.04]	0.01	39%	6	-0.59 [-0.94,-0.25]	0.0007	87%	6	-0.29 [-0.44,-0.15]	<0.0001	29%
<100	8	-0.49 [-0.73,-0.25]	<0.0001	52%	8	-0.57 [-0.89,-0.25]	0.0005	71%	7	-0.26 [-0.56,0.05]	0.1	67%
Region												
Asia	12	-0.37 [-0.54,-0.19]	<0.0001	57%	11	-0.66[-0.94,-0.38]	<0.00001	82%	10	-0.37 [-0.51,-0.24]	<0.00001	24%
Europe	4	-0.16[-0.40,0.08]	0.18	53%	3	-0.30 [-0.51,-0.10]	0.003	0%	3	0.04 [-0.16,0.24]	0.67	0%

HR, Heart rate; SBP, Systolic blood pressure; DBP, Diastolic blood pressure; OR, odds ratio; CI, confidence interval.

## 4 Discussion

With the rapid aging of the global population, the incidence of cataracts is rising significantly, affecting an estimated 95 million people worldwide, making it one of the leading causes of vision impairment and blindness (31). Although cataract surgery is technically advanced, patients are required to remain awake during the procedure, which often leads to anxiety, nervousness, and other negative emotions, as well as some degree of pain. These emotional responses and discomfort may affect the smooth progression of the surgery and postoperative recovery. In recent years, music intervention, as a non-pharmacological and non-invasive therapeutic method, has garnered increasing attention in the medical community. Previous studies have shown that music intervention can have positive effects in alleviating anxiety and pain in surgical patients and stabilizing vital signs (32).

### 4.1 Main findings

This study found that music intervention demonstrated significant therapeutic effects in improving both physiological and psychological health outcomes in cataract surgery patients. Postoperative assessments revealed significant differences in anxiety SAS scores, HR, VAS scores, SBP, DBP, and STAI scores. Sensitivity analysis using the leave-one-out method for SAS score, HR, VAS score, SBP, DBP, and STAI indicated that excluding any single study did not significantly alter the overall results, and the direction of the combined effect remained unchanged, confirming the stability of the pooled results. However, for VAS score, excluding the studies by Guerrier (11) and Xiao (28) changed the result from significant to non-significant, indicating potential instability. Egger’s test revealed publication bias for HR, while no bias was detected for SAS, VAS, SBP, DBP, and STAI scores.

The findings of this study are consistent with those of the systematic review and meta-analysis conducted by Chen et al. (33). This meta-analysis included 15 RCTs with data from 2,098 patients and similarly confirmed the positive effects of music intervention in ophthalmic surgery. The study found that music not only significantly reduced patients’ anxiety and pain levels (p<0.0001), but also moderately improved physiological parameters such as blood pressure. However, this study builds upon Chen et al.’s work by incorporating the most recently published RCTs and specifically focusing on cataract surgery within ophthalmic procedures. This approach helped avoid potential heterogeneity due to differences in treatment methods. The conclusions of this study further validate that music intervention is both cost-effective and safe, providing strong support for improving the perioperative experience of cataract patients. It also confirms the clinical value of music intervention as an adjunctive therapy in cataract surgery.

### 4.2 Regional differences analysis

This study found that the response to music intervention varied between regions, with patients in Europe showing significantly lower efficacy compared to those in Asia. This could be attributed to differences in music intervention methods, types of music, and surgical procedures. In the study by Xiong et al. (29), background music with slow tempos, lyrical melodies, and smooth rhythms was played in the waiting room and operating room. This music was played throughout the patient’s waiting time and surgery, creating a calming environment, with the volume adjusted for patient comfort to avoid interfering with communication between the patient and healthcare providers. This approach effectively reduced anxiety levels, stabilized blood pressure and heart rate, and improved patient satisfaction. In contrast, Bellan et al. (34) had patients listen to music of their own choice, such as classical, country, jazz, or light rock, through headphones during surgery. However, the use of headphones during surgery could have obstructed communication between the patient and medical staff, increasing the patient’s sense of unease. Moreover, differences in musical preferences due to cultural backgrounds, as well as the timing and methods of music intervention, might also influence the effectiveness of the intervention. Therefore, the lower efficacy observed in European patients may be due to differences in the method of music intervention and cultural background. Additionally, the significantly smaller number of studies conducted in Europe compared to Asia could also be a potential factor contributing to the negative results in the European subgroup.

### 4.3 Mechanism of action of music intervention

Music influences brain activity through auditory nerve conduction, regulating the autonomic nervous system function, enhancing parasympathetic nervous activity, and reducing sympathetic nervous tension, thereby producing a calming and relaxing effect (35). Studies have shown that music can stimulate the release of neurotransmitters such as acetylcholine, norepinephrine, and endogenous opioids, which have sedative, mood-enhancing, and emotional-regulating effects (28). These physiological changes help reduce anxiety and fear in patients, stabilize blood pressure and heart rate, and improve surgical satisfaction (29).

In addition, music can divert the patient’s attention away from the surgical environment, reducing sensitivity to operating room noise (such as the sound of phacoemulsification machines) and creating a soothing atmosphere (34). This is especially important for cataract surgery performed under local anesthesia, where the patient remains fully awake throughout the procedure. The patient may be highly sensitive to the injection of local anesthesia, surgical stimuli, instrument handling, and communication between medical staff, making them more prone to anxiety (9). Playing slow-tempo, lyrical background music throughout the surgery can meet both the physiological and psychological needs of the patient, facilitating the smooth progress of the surgery and aiding in recovery (29, 36).

### 4.4 Limitations

This study has several limitations. First, there were significant variations in the types of music interventions and assessment criteria used in the included studies. For example, Xiao et al. (28) assessed anxiety using the anxiety-VAS scale, while Wiwatwongwana et al. (21) and Musa et al. (20) used different assessment tools, leading to high heterogeneity in the results. Although most studies lacked clear descriptions regarding blinding and allocation concealment, as shown in the quality assessment chart, this did not significantly affect our result analysis. Most of the studies were from single-center, small-sample research, and there were notable differences in the personalization of music selection. For instance, Li Na et al. (15).

Second, the subjectivity of pain assessment scales represents another important limitation. As noted by the reviewer, scales such as VAS cannot provide optimal assessment due to their subjective nature, which may lead to different results even within the same patient. This inherent subjectivity of pain measurement tools could contribute to result bias and explain some of the heterogeneity observed between studies. Future research should consider incorporating more objective pain assessment methods or combining subjective scales with physiological parameters to provide a more comprehensive evaluation of pain responses allowed patients to choose their own music types, whereas Xiong Shanjiao et al. (29) used a standardized background music approach. These differences may have led to publication bias and regional selection bias.

Moreover, the influence of cultural background on music preferences was not sufficiently considered in this study. For example, Li Jianzhen et al. (16) demonstrated the effects of Five Elements Zhi Tiao music in Chinese patients, which may differ from the effects of binaural beat music, as studied by Loong et al. (19) in patients from other cultural backgrounds. Evidence regarding the optimal timing of music interventions remains limited. While Guerrier et al. (11) explored the effects of preoperative music intervention, there was a lack of systematic comparative studies between preoperative, intraoperative, and postoperative music interventions.

Therefore, further multi-center, large-scale, high-quality randomized controlled trials are needed to improve the level of evidence and provide more reliable guidance for clinical practice.

## 5 Conclusion

This study found that music intervention can significantly improve SAS, HR, VAS, SBP, DBP, and STAI scores in cataract surgery patients. Subgroup analysis suggested that the efficacy of music intervention was better in patients from Asia compared to those from Europe. Given the limitations of this study, such as small sample size, potential heterogeneity, and instability, further large-scale, multi-center RCTs are needed to confirm the effects of music intervention on cataract surgery patients.

## Data Availability

The original contributions presented in the study are included in the article/**Supplementary Material**. Further inquiries can be directed to the corresponding author.
