# Supplementary material for: Efficacy of music intervention on pain and anxiety in patients undergoing cataract surgery: a systematic review and meta-analysis
Source: Front Psychiatry. 2025 Jun 19;16:1600359. doi: 10.3389/fpsyt.2025.1600359 (PMC12222141; doi:10.3389/fpsyt.2025.1600359)
Supplement: Supplementary file 1 [file DataSheet1.docx]

**TableS1 Literature Search Strategy**

1. Pubmed-266

**((((((((((((((((Surgical Procedures, Operative) OR (Operative Procedures)) OR (Operative Procedure)) OR (Procedure, Operative)) OR (Procedures, Operative)) OR (Surgical Procedure, Operative)) OR (Operative Surgical Procedures)) OR (Procedure, Operative Surgical)) OR (Procedures, Operative Surgical)) OR (Surgical Procedures)) OR (Procedure, Surgical)) OR (Procedures, Surgical)) OR (Surgical Procedure)) OR (Operative Surgical Procedure)) OR (Surgery)) AND ((((((((((((Cataract) OR (Cataracts)) OR (Lens Opacities)) OR (Lens Opacity)) OR (Opacities, Lens)) OR (Opacity, Lens)) OR (Cataract, Membranous)) OR (Cataracts, Membranous)) OR (Membranous Cataract)) OR (Membranous Cataracts)) OR (Pseudoaphakia)) OR (Pseudoaphakias))) AND (((((((((((((((((Music) OR (Rap Music)) OR (Music, Rap)) OR (Hip Hop Music)) OR (Hop Music, Hip)) OR (Music, Hip Hop)) OR (Jazz Music)) OR (Music, Jazz)) OR (Classical Music)) OR (Music, Classical)) OR (Songs)) OR (Song)) OR (Vocal Melody)) OR (Melodies, Vocal)) OR (Melody, Vocal)) OR (Vocal Melodies)) OR (Rock and Roll Music))**

2. Embase-442

**((((((((((((((((Surgical Procedures, Operative) OR (Operative Procedures)) OR (Operative Procedure)) OR (Procedure, Operative)) OR (Procedures, Operative)) OR (Surgical Procedure, Operative)) OR (Operative Surgical Procedures)) OR (Procedure, Operative Surgical)) OR (Procedures, Operative Surgical)) OR (Surgical Procedures)) OR (Procedure, Surgical)) OR (Procedures, Surgical)) OR (Surgical Procedure)) OR (Operative Surgical Procedure)) OR (Surgery)) AND ((((((((((((Cataract) OR (Cataracts)) OR (Lens Opacities)) OR (Lens Opacity)) OR (Opacities, Lens)) OR (Opacity, Lens)) OR (Cataract, Membranous)) OR (Cataracts, Membranous)) OR (Membranous Cataract)) OR (Membranous Cataracts)) OR (Pseudoaphakia)) OR (Pseudoaphakias))) AND (((((((((((((((((Music) OR (Rap Music)) OR (Music, Rap)) OR (Hip Hop Music)) OR (Hop Music, Hip)) OR (Music, Hip Hop)) OR (Jazz Music)) OR (Music, Jazz)) OR (Classical Music)) OR (Music, Classical)) OR (Songs)) OR (Song)) OR (Vocal Melody)) OR (Melodies, Vocal)) OR (Melody, Vocal)) OR (Vocal Melodies)) OR (Rock and Roll Music))**


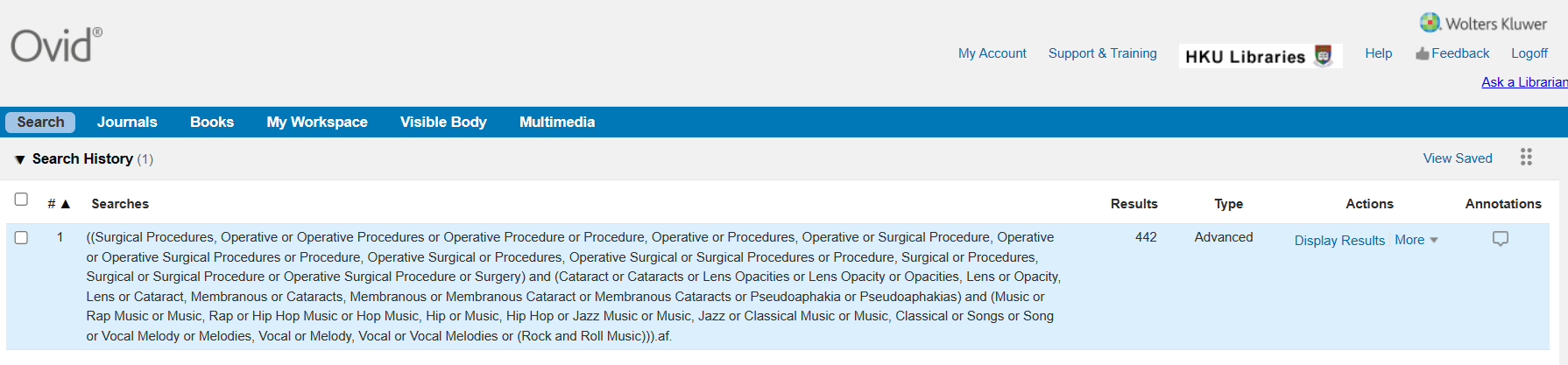


3. Cochrane-72


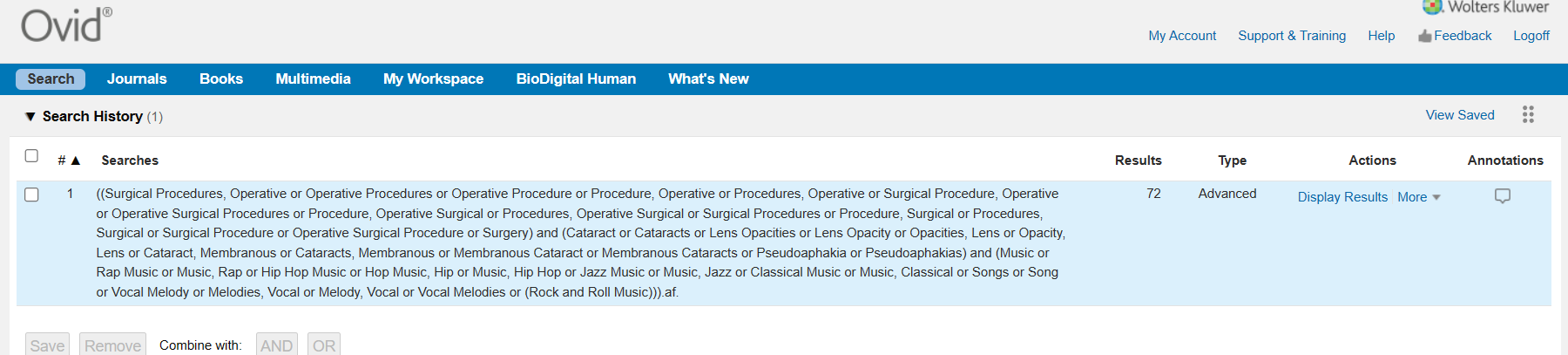


4. web of science -34

**((((((((((((((((Surgical Procedures, Operative) OR (Operative Procedures)) OR (Operative Procedure)) OR (Procedure, Operative)) OR (Procedures, Operative)) OR (Surgical Procedure, Operative)) OR (Operative Surgical Procedures)) OR (Procedure, Operative Surgical)) OR (Procedures, Operative Surgical)) OR (Surgical Procedures)) OR (Procedure, Surgical)) OR (Procedures, Surgical)) OR (Surgical Procedure)) OR (Operative Surgical Procedure)) OR (Surgery)) AND ((((((((((((Cataract) OR (Cataracts)) OR (Lens Opacities)) OR (Lens Opacity)) OR (Opacities, Lens)) OR (Opacity, Lens)) OR (Cataract, Membranous)) OR (Cataracts, Membranous)) OR (Membranous Cataract)) OR (Membranous Cataracts)) OR (Pseudoaphakia)) OR (Pseudoaphakias))) AND (((((((((((((((((Music) OR (Rap Music)) OR (Music, Rap)) OR (Hip Hop Music)) OR (Hop Music, Hip)) OR (Music, Hip Hop)) OR (Jazz Music)) OR (Music, Jazz)) OR (Classical Music)) OR (Music, Classical)) OR (Songs)) OR (Song)) OR (Vocal Melody)) OR (Melodies, Vocal)) OR (Melody, Vocal)) OR (Vocal Melodies)) OR (Rock and Roll Music))** (主题)


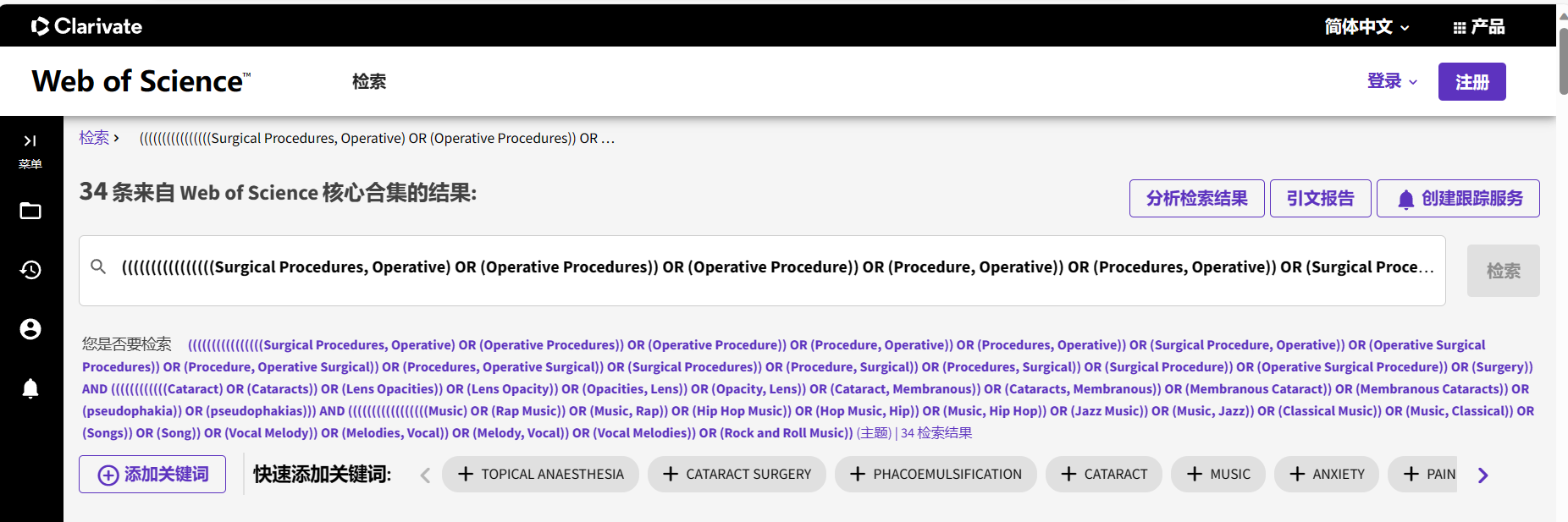


5. Wanfang-23


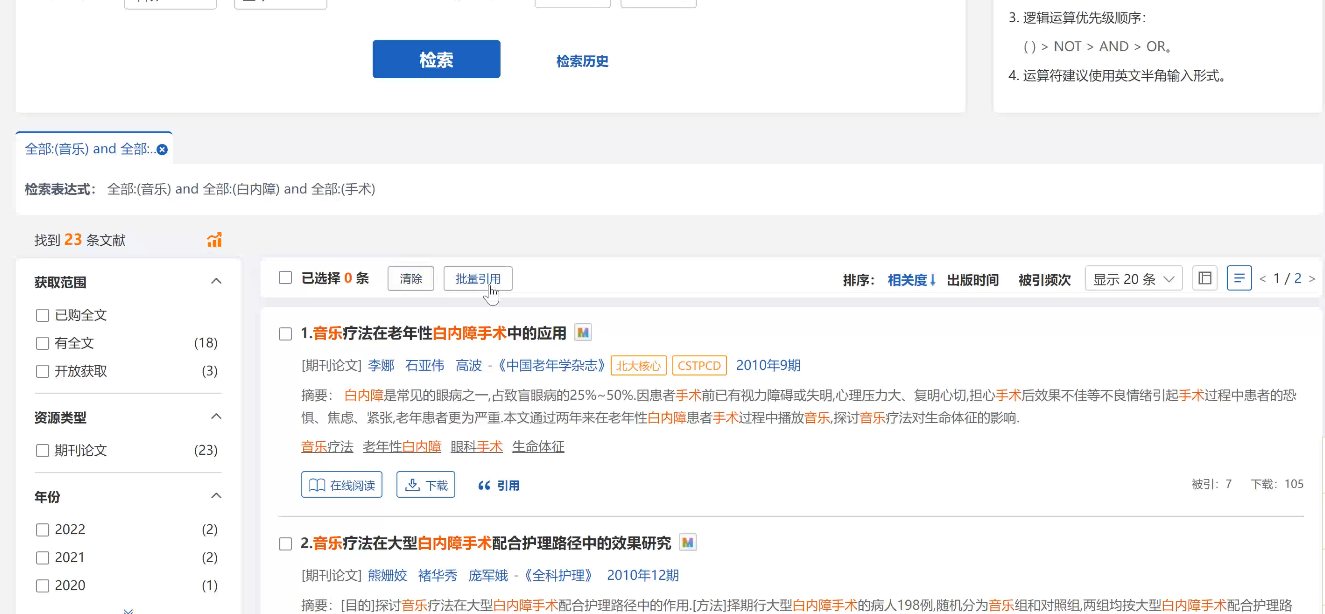


6. Cnki-19


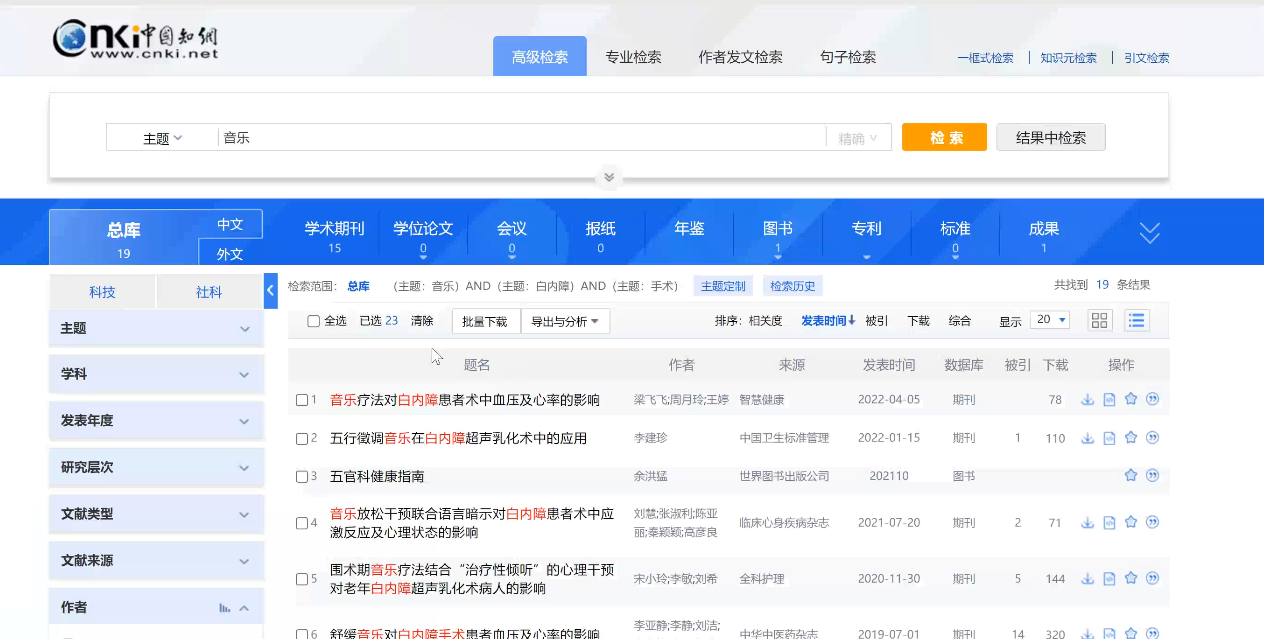


**Surgical Procedures, Operative/ Operative Procedures/Operative Procedure/Procedure, Operative/Procedures, Operative/Surgical Procedure, Operative/Operative Surgical Procedures/Procedure, Operative Surgical/Procedures, Operative Surgical/Surgical Procedures/Procedure, Surgical/Procedures, Surgical/Surgical Procedure/Operative Surgical Procedure/Surgery/Cataract/Cataracts/Lens Opacities/Lens Opacity/Opacities, Lens/ Opacity, Lens/Cataract, Membranous/Cataracts, Membranous/Membranous Cataract/Membranous Cataracts/Pseudoaphakia/Pseudoaphakias/Music/Rap Music/ Music, Rap/Hip Hop Music/Hop Music, Hip/Music, Hip Hop/Jazz Music/Music, Jazz/Classical Music/Music, Classical/Songs/Song/Vocal Melody/Melodies, Vocal/Melody, Vocal/Vocal Melodies/Rock and Roll Music。**
